# Event-triggered adaptive sliding mode control for consensus of multiagent systems with unknown disturbances

**DOI:** 10.1038/s41598-022-21704-4

**Published:** 2022-10-19

**Authors:** Tianxing Chen, Xuebin Zhuang, Zhiwei Hou, Hongbo Chen

**Affiliations:** School of Systems Science and Engineering, Sun Yat-Sen University, Guangzhou, 510000 China

**Keywords:** Engineering, Aerospace engineering

## Abstract

In this paper, a novel robust distributed consensus control scheme based on event-triggered adaptive sliding mode control is proposed for multiagent systems with unknown disturbances in a leader-follower framework. First, an adaptive multivariate disturbance observer is utilized to compensate for the disturbance of each agent. Next, a distributed consensus control protocol is constructed via integral sliding mode control, in which a novel adaptive law is designed for the switching gain to overcome the unknown perturbations. An event-triggered strategy is designed to update the control input. Furthermore, the feasibility of the proposed scheme is rigorously analyzed by Lyapunov theory, and a lower bound expression for the inter-event time is derived to guarantee that Zeno behavior can be excluded. The proposed nonlinear consensus algorithm is remarkable in that it does not require any information about the bounds of the disturbances. Finally, compared with existing methods, the proposed algorithm is validated through detailed numerical simulations. In addition, the proposed algorithm is applied to a group of UAVs in this paper, and the results show that it has more application value.

## Introduction

In recent years, the consensus of distributed cooperative control has become an extremely promising research area for multiagent systems (MASs) due to its potential applications in attitude synchronization of satellites^1^, mobile robots/unmanned aerial vehicles (UAVs)^2–4^ and microgrids^5^. The MAS is a cyber-physical system consisting of physical and information-driven functions^6,7^. In this type of system, it is the recent trend to use digital platforms to make control system and physical system connected^8^. However, digital implementation may lead to other problems, such as limited onboard energy and how to determine the frequency of control signals to guarantee system stability^7^. In addition, the existence of unknown disturbances in MASs would make the problem of distributed consensus control more complicated.

Many related works have intensively investigated the problem of consensus control for MASs, such as the first-order/second-order systems^9,10^, heterogeneous systems^11,12^ and consensus in communication delay^13,14^ or homologous attack^15,16^. Some related studies further focus on the uncertainties in the system. In order to achieve consensus tracking of MASs with no-identical dynamics, a new compensation term^17^ is designed to offset the effects caused by the uncertainties. A distributed adaptive consensus tracking controller proposed by Wang^18^ can further relax the assumption of linearly expressible reference trajectories^17^. Moreover, there are also many related studies on MASs with Multi-input Multi-output (MIMO) dynamics, such as mobile robots/UAVs^19–22^. Zhang^19^ designs a consensus algorithm for a class of nonlinear multi-vehicle systems in strict feedback form. A semi-global leader-following consensus-based approach is proposed by Zhou^20^ to achieve consensus formation of a group of UAVs, with both the leader and the followers subject to input saturation. To achieve distributed time-varying formation tracking control of quadrotors, an extended state observer^22^ is designed to enhance the anti-disturbance capability of the quadrotor. Generally, the control signals obtained by the above methods are continuous in time, but in practice they are sampled and implemented using digital platforms. So the value of sampling period is crucial, namely not too small to cause excessive control expenditures^23,24^ and not too large to guarantee control performance, which these methods may be difficult to determine accurately.

In order to deal with this issue, event-triggered control algorithms have been proposed^25,26^. The idea of event-triggered control is to abandon the paradigm of periodic sampling control, and the control signals are updated only when needed. This means that it is an energy-efficiency solution that can effectively improve control efficiency while guaranteeing control performance^27,28^. Owing to this advantage, event-triggered control has been widely used to solve the consensus control problems of the MASs in the past few years^28^. Different triggering mechanisms have been developed, such as self-trigger^29^, distributed trigger^30^, and dynamic event-trigger^31,32^. In addiction, considering the disturbances in the system, achieving a robust performance is also one of the main concerns for the design of the event-triggered consensus controller^33^.

Sliding mode control (SMC) is a very popular robust control technique that can effectively handle bounded disturbances and uncertainties. Behera^33^ shows that event-triggered SMC still has great potential in solving uncertainties. In order to achieve time-varying formation control for the high-order MASs with external disturbances, an event-triggered SMC consensus approach is proposed by Wang^34^. Cui^35^ and Wang^36^ respectively develop a distributed finite-time consensus algorithm based on event-triggered SMC for the second-order MAS with disturbances, and the major difference between their algorithms is the choice of trigger mechanism. Nair^37^ and Nandanwar^38^ respectively design a novel event-triggered scheme based on SMC for the consensus-based tracking control of MIMO multirobotic systems with disturbances, and the major difference between their algorithms lies in the choice of the types of SMC. One limitation of the methods aforementioned is that the upper boundary of disturbance has to be known in advance. Adaptive Sliding Mode Control (ASMC) aims to adapt the switching gain in such a way to cope with possibly unknown uncertainty. However, there is less literature about the application of the event-triggered ASMC on the consensus control of MASs. It is worth mentioning that an event-triggered ASMC algorithm is proposed by Li^39^ for a class of Takagi-Sugen fuzzy systems with actuator faults and signal quantization. This indicates that event-triggered ASMC has great potential for handling uncertainties in the system.

In contrast to periodic/continuous sampling control, event-triggered control does not constantly monitor the state of an already-stable system. Instead, it can check the system state more efficiently to determine when the control input should be updated. This means that event-triggered control can increase efficiency while guaranteeing control performance. In practical applications, considering the limited onboard energy, especially for small mobile robots/UAVs, it is necessary to develop an energy-efficiency robust control algorithm, which constitutes the first motivation for the present article. On the other hand, some recent ASMC methods are able to provide some promising control performance through continuous control signals, such as complete robustness for MASs with unknown bounded disturbances^40^. Therefore, it is necessary to explore the value of event-triggered ASMC for applications in MASs with unknown disturbances, which constitutes the second motivation for the present article.

With the mentioned motivations in mind, this paper aims at developing a distributed robust consensus control scheme based on event-triggered ASMC for consensus tracking of MASs with unknown disturbances, and investigate the advantages of this algorithm over existing ones. The main contributions of this paper are highlighted as follows

1. A novel robust distributed consensus control algorithm based on event-triggered ASMC is developed for the MIMO MASs with uncertain disturbances in a leader-follower framework. Compared with algorithm^37,38^, the proposed algorithm does not require any information on the bounds of the disturbances.
2. The feasibility of the proposed algorithm is rigorously analyzed by Lyapunov theory, and a lower bound expression for the inter-event time is derived to guarantee that Zeno behavior can be excluded.
3. Compared with existing methods, the proposed algorithm is validated through detailed numerical simulations. In addition, the proposed algorithm is applied to a group of UAVs in this paper, and the results show that it has more application value than algorithm^37^.

The remainder of this paper is structured as follows. Problem formulation and some useful lemmas are described in “Problem formulation” section. The consensus algorithm based event-triggered ASMC is developed in “Event-triggered ASMC based consensus algorithm” section, simulation and experimental validation results of the proposed algorithm are given in “Results” section. Finally, conclusions and future works are stated in “Conclusion” section.

*Notation* Let ${\mathbb {R}}$ and ${\mathbb {R}}^n$ denote the set of real numbers and n-dimensions column vectors, respectively. ${{\mathbf{x}}^T}$ denotes the transpose of the matrix or vector ${{\mathbf{x}}}$. $\left\| {\left( \cdot \right) } \right\|$ and ${\lambda _{\min }}\left( \cdot \right)$ represent 2-norm and the smallest eigenvalue of $\left( \cdot \right)$ , respectively. ${{\mathbf{I}}_n}$ is the identity matrix having n-dimensions and $\otimes$ stands for Kronecker product for matrices.

## Problem formulation

Consider a MAS composed of *N* members. The communication topology among the agents can be described by a directed graph ${G=(V,E,A)}$, where the set of nodes is denoted by ${V=(V_1,V_2...V_N)}$, and the set of edges is ${E\subseteq V \times V}$. Let $A = \left[ {{a_{ij}}} \right]$ denote the weighted adjacency matrix, in which ${a_{ij}} > 0$, if and only if $\left( {{V_i},{V_j}} \right) \subseteq E$, otherwise ${a_{ij}} = 0$. For any ${V_i}$, the neighbor set is given by ${N_i} = \left\{ {{V_j}:\left( {{V_i},{V_j}} \right) \subseteq E} \right\}$. The Laplacian is represented by $L = D - A$, where $D = {\mathrm{diag}}\left\{ {{d_1},{d_2},...{d_N}} \right\}$ is the degree matrix, and ${d_i} = \sum \nolimits _{j = 1}^n {{a_{ij}}}$.

For a MAS based on the leader-follower framework, with one leader and *N* followers, the leader is represented by a node ${V_0}$ and the follower is represented by $\left\{ {{V_1},{V_2},{V_3}...{V_N}} \right\}$. The connection weight matrix of the leader-follower framework is defined as $B = diag\left\{ {{b_1},{b_2},...{b_N}} \right\}$, ${b_i} > 0$, if and only if the leader is connected to the ${i^{th}}$ follower, otherwise ${b_i} = 0$.

The MIMO dynamics of leader and followers in MAS framework can be described as follows

1 $$\begin{aligned} \begin{aligned} {\dot{\mathbf{x}}}_i\left( t \right) &={\mathbf{u}}_i\left( t \right) + {\mathbf{d}}_i\left( t \right) , i = 1...N\\ {\dot{\mathbf{x}}}_0\left( t \right) &={\mathbf{u}}_0\left( t \right) \end{aligned} \end{aligned}$$

where ${{{\mathbf{x}}}_i\left( t \right) } \in {\mathbb {R}}^n, {{{\mathbf{u}}}_i\left( t \right) } \in {\mathbb {R}}^n, {{{\mathbf{d}}}_i\left( t \right) } \in {\mathbb {R}}^n$ represent the state vector, the control input and the unknown disturbances for the ${i^{th}}$ follower, respectively; ${{{\mathbf{x}}}_0\left( t \right) } \in {\mathbb {R}}^n$ and ${{{\mathbf{u}}}_0\left( t \right) } \in {\mathbb {R}}^n$ respectively represent the leader’s state vector and control input, and the leader is considered an ideal reference without any disturbance. Let ${\tilde{\mathbf{x}} }_i\left( t \right) = {\mathbf{x}}_i\left( t \right) - {\mathbf{x}}_0\left( t \right) + {\varvec{\delta } }_i$, ${\tilde{\mathbf{u}} }_i\left( t \right) = {\mathbf{u}}_i\left( t \right) - {\mathbf{u}}_0\left( t \right)$ be the deviations in state and control input of the ${i^{th}}$ follower from the leader, respectively. ${{\varvec{\delta } }_i} \in {\mathbb {R}}^n$, a design vector that can be arbitrarily designed, is the desired state deviation of the ${i^{th}}$ follower from leader. The relative dynamics of agent *i* based on the above deviations and (1) can further be described as

2 $$\begin{aligned} \begin{aligned} {{\dot{\tilde{\mathbf{x}}}}}_i(t) = {\tilde{\mathbf{u}}}_i\left( t \right) + {\mathbf{d}}_i\left( t \right) \end{aligned} \end{aligned}$$

For the agent dynamics (2), the unmodeled dynamics and external disturbances/uncertainties lumped together as ${{\mathbf{d}}_i\left( t \right) }$ is assumed to be bounded, let us consider the state-dependent upper bound as^41^

3 $$\begin{aligned} \begin{aligned} \left\| {{{\mathbf{d}}_i}\left( t \right) } \right\| \le \sum \limits _{j = 0}^2 {K_j^{*}{{\left\| {{{{\tilde{\mathbf{x}}}}_i}\left( t \right) } \right\| }^j}} ,\forall t \ge 0 \end{aligned} \end{aligned}$$

with unknown parameters $K_j^{*} > 0,j = 0,1,2$. Note that (3) only considers that the upper bound fits a state-dependent upper bound structure and does not impose a priori bounds on the unknown disturbances.

In this paper, our objective is to develop a robust distributed consensus control algorithm and design an event-triggered strategy to update the control input such that each follower can asymptotically follow the leader’s trajectory, in spite of the unknown disturbances. It should be noted that in (3), the unknown disturbances ${{{\mathbf{d}}}_i\left( t \right) }$ may have the following constraints: (i) no a priori constant upper bound; (ii) no certain compositional structure. These constraints make the control algorithm design more challenging, which is also the main challenge in this paper. Some useful lemmas may be used in the following analysis.

### Lemma 1

^27,37,42^: For the MAS, its communication topology graph *G* contains a directed spanning tree, then all the eigenvalues of $L + B$ have positive real parts.

### Lemma 2

^27,42^: For ${\mathbf{x}}_i \in {\mathbb {R}}^n, i = 1...n$ and $\alpha \in \left( {0,1} \right]$, then ${\left( {\sum \nolimits _{i = 1}^n {\mid {{x_i}} \mid } } \right) ^\alpha } \le {\sum \nolimits _{i = 1}^n {\mid {{x_i}} \mid } ^\alpha } \le {{n}^{1 - \alpha }}{\left( {\sum \nolimits _{i = 1}^n {\mid {{x_i}} \mid } } \right) ^\alpha }$, for $\mid \alpha \mid \in \left( {0,1} \right)$, $\left\| {{\mathbf{x}}_i^\alpha } \right\| \le {n^{1 - \alpha }}{\left\| {{{\mathbf{x}}_i}} \right\| ^\alpha }$.

The event-triggered ASMC based consensus algorithm is detailed in the next section.

## Event-triggered ASMC based consensus algorithm

### Disturbance observer

In this paper, inspired by Tian^43^, the following adaptive multivariable disturbance observer is utilized to compensate the disturbances ${\mathbf{d}}_i\left( t \right)$ in the MAS described by (2), in order to enhance the robustness of the control algorithm. The adaptive multivariable disturbance observer can be described as

4 $$\begin{aligned} \begin{aligned} {{{\dot{\hat{\mathbf{x}}}}}_i}\left( t \right)&= - {k_1}\left( t \right) \frac{{{{\mathbf{E}}_i}\left( t \right) }}{{{{\left\| {{{\mathbf{E}}_i}\left( t \right) } \right\| }^{{1 / 2}}}}} - {k_2}\left( t \right) {{\mathbf{E}}_i}\left( t \right) + {{{\hat{\mathbf{d}}}}_i}\left( t \right) + {{{\tilde{\mathbf{u}}}}_i}\left( t \right) \\ {{{\dot{\hat{\mathbf{d}}}}}_i}\left( t \right)&= - {k_3}\left( t \right) \frac{{{{\mathbf{E}}_i}\left( t \right) }}{{\left\| {{{\mathbf{E}}_i}\left( t \right) } \right\| }} - {k_4}\left( t \right) {{\mathbf{E}}_i}\left( t \right) \end{aligned} \end{aligned}$$

where ${\hat{\mathbf{d}}}_i\left( t \right)$ and ${{{\hat{ \mathbf{x}}}}_i}\left( t \right)$ represent the estimation results of ${\mathbf{d}}_i\left( t \right)$ and ${{\mathbf{x}}_i}\left( t \right)$ in (2), respectively. The state estimation error ${{\mathbf{E}}_i}\left( t \right) = {{{{{\hat{\mathbf{x}}}}}}_i}\left( t \right) - {{\mathbf{x}}_i}\left( t \right)$ and ${{\tilde{\mathbf{u}}}_i}\left( t \right)$ is the system control input, which will be designed in (13). The adaptive gains ${k_w}\left( t \right) \left( {w = 1,2,3,4} \right)$ are designed as

5 $$\begin{aligned} \begin{aligned} {k_1}\left( t \right)&= {c_1}{G^{{1 / 2}}}\left( t \right) ,{k_2}\left( t \right) = {c_2}G\left( t \right) ,{k_3}\left( t \right) = {c_3}G\left( t \right) \\ {k_4}\left( t \right)&= {c_4}{G^2}\left( t \right) ,\dot{G}\left( t \right) = \left\| {{{\mathbf{E}}_i}\left( t \right) } \right\| - {\alpha _g}G\left( t \right) \end{aligned} \end{aligned}$$

where ${\alpha _g}$ is a positive design scalar, $G\left( t \right)$ represents the increased rate of adaptive gain and initial value $G\left( 0 \right)$ is a positive constant, the parameters ${c_w}\left( {w = 1,2,3,4} \right)$ are positive constants and satisfy the following conditions

6 $$\begin{aligned} \begin{aligned} 9{c_1^2}{c_2^2} + 8{c_2^2}{c_3} < 4{c_3}{c_4} \end{aligned} \end{aligned}$$

### ASMC based consensus

In the absence of unknown disturbances ${\mathbf{d}}_i\left( t \right)$, the system (2) can achieve consensus tracking, if the following control protocol is chosen as follow^27,37,38^

7 $$\begin{aligned} \begin{aligned} {\tilde{\mathbf{u}} }_i\left( t \right) &={\mathbf{q}}_i^\eta \left( t \right) \\ {\mathbf{q}}_i \left( t \right) &=- \frac{{{\mu _i}}}{{{n_i} + 1}}\left\{ {\sum \limits _{j \subseteq {N_i}} {{a_{ij}}\left[ {\left( {{{\mathbf{x}}_i}\left( t \right) - {{\mathbf{x}}_0}\left( t \right) + {{{\varvec{\delta } }}_i}} \right) - \left( {{{\mathbf{x}}_j}\left( t \right) - {{\mathbf{x}}_0}\left( t \right) + {{{\varvec{\delta } }}_i}} \right) } \right] + {b_i}\left( {{{\mathbf{x}}_i}\left( t \right) - {{\mathbf{x}}_0}\left( t \right) + {{{\varvec{\delta } }}_i}} \right) } } \right\} \\ &=- \frac{{{\mu _i}}}{{{n_i} + 1}}\left( {\sum \limits _{j \subseteq N_i} {{a_{ij}}\left( {{{{{{\tilde{\mathbf{x}}}}}}_i}\left( t \right) - {{{{{\tilde{\mathbf{x}}}}}}_j}\left( t \right) } \right) + {b_i}{{{\tilde{\mathbf{x}}}}_i}\left( t \right) } } \right) \end{aligned} \end{aligned}$$

where $\eta \in \left( {0.5,1} \right)$ is strictly the ratio of positive odd numbers, $1 \le {n_i} \le N$ is the number of neighboring agents for the ${i^{th}}$ follower and ${\mu _i}$ is a positive design scalar. For the design scalars ${a_{ij}}$, ${b_i}$ and ${{\varvec{\delta }}_i}$ are the basic parameters of the formation, which are described in detail in Section 2.

Considering the disturbances in the system, the above approach can be improved by introducing integral sliding mode control. The integral type sliding surface is defined as

8 $$\begin{aligned} \begin{aligned} {\mathbf{S}}_i\left( t \right) = {\tilde{\mathbf{x}}}_i\left( t \right) - \int _0^t {{\mathbf{q}}_i^\eta \left( t \right) dt} ,i = 1...N \end{aligned} \end{aligned}$$

where ${{\mathbf{S}}_{\mathrm{{i}}}}\left( {\mathrm{t}} \right) = {\left[ {{s_1}\left( t \right) ,{s_2}\left( t \right) ,...{s_n}\left( t \right) } \right] ^T}$. For faster convergence, we have chosen a fast reaching law^37^ given by

9 $$\begin{aligned} \begin{aligned} {\dot{\mathbf{S}}}_i\left( t \right) = - {{\rho }}\left( t \right) {\mathbf{sign}}\left( {{{\mathbf{S}}_i}\left( t \right) } \right) - {\varvec{\Lambda }}{{\mathbf{S}}_i}\left( t \right) \end{aligned} \end{aligned}$$

where ${\mathbf{sign}}\left( {{{\mathbf{S}}_i}\left( t \right) } \right) = \left[ {{\mathrm{sign}}\left( {{s_1}\left( t \right) } \right) ,{\mathrm{sign}}\left( {{s_2}\left( t \right) } \right) ,...{\mathrm{sign}}\left( {{s_n}\left( t \right) } \right) } \right] ^T$, and the user-defined gain matrix ${\varvec{\Lambda }} = {\mathrm{diag}}{\left[ {{\Lambda _1},{\Lambda _2},...{\Lambda _n}} \right] }$ with each of its elements being positive gains. Inspired by Spandan^41^, the adaptive law for the switching gain ${{\rho }_i}\left( t \right)$ can be designed as

10 $$\begin{aligned} \begin{aligned} {{\rho }_i}\left( t \right) = \sum \nolimits _{j = 0}^2 {{{K}_j\left( t \right) }{{\left\| {{{ \tilde{\mathbf{x}} }_i}\left( t \right) } \right\| }^j}} + {{\tau }_i} \end{aligned} \end{aligned}$$

where ${{\tau }_i}$ is a positive design constant. The gains ${{K}_j \left( t \right) }$ are adapted via

11 $$\begin{aligned} \begin{aligned} {{\dot{K}}}_j \left( t \right) = \left\| {{{\mathbf{S}}_i}\left( t \right) } \right\| {\left\| {{{\tilde{\mathbf{x}}}_i}\left( t \right) } \right\| ^j} - {\alpha _j}{K}_j \left( t \right) \text {with } {K}_j \left( 0 \right)> 0,{\alpha _j} > 0 \end{aligned} \end{aligned}$$

where ${\alpha _j} \in {\mathbb {R}}^+ ,j = 0,1,2$ are design scalars.

Hence, the ASMC based consensus algorithm can be defined as

12 $$\begin{aligned} \begin{aligned} {\tilde{\mathbf{u}}}_i\left( t \right) = {\mathbf{q}}_i^\eta \left( t \right) - {{\rho }_i}\left( t \right) {\mathbf{sign}}\left( {{{\mathbf{S}}_i}\left( t \right) } \right) - {{\varvec{\Lambda }}}{{\mathbf{S}}_i}\left( t \right) \end{aligned} \end{aligned}$$

Moreover, the disturbance’s estimation results ${\hat{\mathbf{d}}}_i\left( t \right)$ in (4) can be used to compensate the disturbances ${{{\mathbf{d}}}_i\left( t \right) }$ in the system, in order to enhance the robustness of the control algorithm. Similar works we can be found in^17,18,22,43^. Therefore, (12) can be further described as

13 $$\begin{aligned} \begin{aligned} {\tilde{\mathbf{u}} }_i\left( t \right) &={\mathbf{q}}_i^\eta \left( t \right) - {{\rho }_i}\left( t \right) {\mathbf{sign}}\left( {{{\mathbf{S}}_i}\left( t \right) } \right) - {\varvec{\Lambda }}{{\mathbf{S}}_i}\left( t \right) - {\hat{\mathbf{d}}}_i\left( t \right) \end{aligned} \end{aligned}$$

### Design the event-triggered strategy

In this paper, an event-triggered strategy is designed for the ASMC based consensus algorithm to update the control input, which can be described as

14 $$\begin{aligned} \begin{aligned} {\tilde{\mathbf{u}}}_i\left( t \right) = {\mathbf{q}}_i^\eta \left( {t_k^i} \right) - {{\rho }_i}\left( {t_k^i} \right) {\mathbf{sign}}\left( {{{\mathbf{S}}_i}\left( {t_k^i} \right) } \right) - {\varvec{\Lambda }}{{\mathbf{S}}}\left( {t_k^i} \right) - {\hat{\mathbf{d}}}_i\left( {t_k^i} \right) \end{aligned} \end{aligned}$$

where $t \in \left[ {t_k^i,t_{k + 1}^i} \right)$ and ${t_k^i}$ is the triggering time.

The measurement error of the event-triggered control strategy is designed as

15 $$\begin{aligned} \begin{aligned} {{\mathbf{e}}_i}\left( t \right) &={\mathbf{q}}_i^\eta \left( {t_k^i} \right) - {{\rho }_i}\left( {t_k^i} \right) {\mathbf{sign}}\left( {{{\mathbf{S}}_i}\left( {t_k^i} \right) } \right) - {\varvec{\Lambda }}{{\mathbf{S}}_i}\left( {t_k^i} \right) - {\hat{\mathbf{d}}}_i\left( {t_k^i} \right) - \left[ {\mathbf{q}}_i^\eta \left( t \right) - {{\rho }_i}\left( t \right) {\mathbf{sign}}\left( {{{\mathbf{S}}_i}\left( t \right) } \right) - {\varvec{\Lambda }}{{\mathbf{S}}_i}\left( t \right) - {\hat{\mathbf{d}}}_i\left( t \right) \right] \end{aligned} \end{aligned}$$

The following triggering condition is defined to determine the inter-event time for the ${i^{th}}$ follower

16 $$\begin{aligned} t_{k + 1}^i = \mathop {\max }\limits _{r \ge t_k^i} \left\{ {r:{f_i}\left( t \right) > 0,\forall t \in \left[ {t_k^i,r} \right] } \right\} \end{aligned}$$

where $${f_i}\left( t \right) = \left\| {{{\mathbf{e}}_i}\left( t \right) } \right\| - {\tau }_i$$.

**Figure 1:**
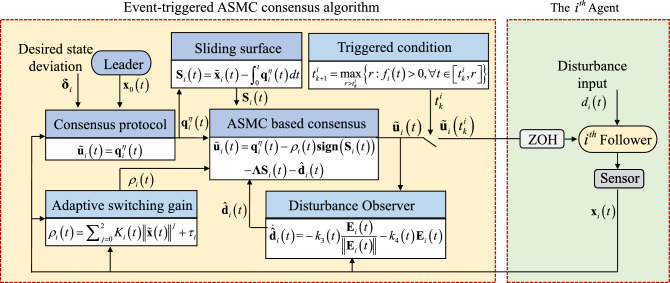
The structure of proposed event-triggered ASMC based consensus algorithm.

Collectively, the algorithmic scheme proposed in this paper constructs an ASMC based consensus algorithm capable of handling unknown disturbances efficiently. The compensation of the disturbance observer can further enhance the robustness of the algorithm. The event-triggered strategy is designed such that the control input of each agent is updated only when the triggering condition is satisfied and remains constant through the Zero-Order Holder (ZOH)^6^ during the inter-event time. The structure of the proposed scheme is shown in Fig. 1.

### Stability analysis

In this subsection, we establish our main theorems and their proofs, and prove theoretically that our proposed scheme is feasible. In the following, the closed-loop stability is analyzed via the notion of Globally Uniformly Ultimately Bounded (GUUB) solutions^44^.

#### Theorem 1

Consider the MAS (2) with control algorithm (14), the communication topology is defined by a connected graph *G* and the triggering condition is given by (16). Then the closed-loop systems are GUUB, and an ultimate bound ${\omega }$ on ${\mathbf{S}}_i\left( t \right)$ is given by

17 $$\begin{aligned} \begin{aligned} \omega = \sqrt{\frac{{\sum \nolimits _{j = 0}^2 {{\alpha _j}{{K}_j^{*}}^2} }}{{{\sigma - \kappa }}}} \end{aligned} \end{aligned}$$

where $\sigma \buildrel \Delta \over = 2{\min _j}\left\{ {{\lambda _{\min }}\left( {\varvec{\Lambda }} \right) ,{{{\alpha _j}} / 2}} \right\}$ and the scalar $\kappa$ satisfy $0< \kappa < \sigma$.

#### Proof

From the laws (11), we can find

18 $$\begin{aligned} \begin{aligned} {K}_j \left( t \right) &=\underbrace{\exp \left( { - {\alpha _j}t} \right) {K}_j\left( 0 \right) }_{ \ge 0} + \underbrace{\int _0^t {\left( { - {\alpha _j}\left( {t - \theta } \right) } \right) } \left( {\left\| {{{\mathbf{S}}_i}\left( \theta \right) } \right\| {{\left\| {{{\tilde{ \mathbf{x}} }_i}\left( \theta \right) } \right\| }^j}} \right) d\theta }_{ \ge 0} \end{aligned} \end{aligned}$$

so it can be concluded that ${K}_j \left( t \right) \ge 0,j = 0,1,2.\forall t \ge 0$ and ${\rho _i}\left( t \right) \ge {\tau _i}$.

It can be found in control algorithm (14) that the unknown disturbances ${{{\mathbf{d}}}_i\left( t \right) }$ are compensated by disturbance observer ${\hat{\mathbf{d}}}_i\left( t \right)$. Thus, (3) can be further described as

19 $$\begin{aligned} \begin{aligned} \left\| {{{\mathbf{d}}_i}\left( t \right) - {{\hat{\mathbf{d}}}_i}\left( t \right) } \right\| \le \sum \limits _{j = 0}^2 {K_j^{*}{{\left\| {{{{{{\tilde{\mathbf{x}}}}}}_i}\left( t \right) } \right\| }^j}} ,\forall t \ge 0 \end{aligned} \end{aligned}$$

Consider the following Lyapunov function candidate

20 $$\begin{aligned} \begin{aligned} V = \frac{1}{2}{\mathbf{S}}_i^T\left( t \right) {{\mathbf{S}}_i}\left( t \right) + \sum \nolimits _{j = 0}^2 {\frac{1}{2}{{\left( {{K}_j \left( t \right) - {K}_j^{*}} \right) }^2}} \end{aligned} \end{aligned}$$

and we can further find

21 $$\begin{aligned} \begin{aligned} {\dot{\mathbf{S}}}_i\left( t \right) &={\dot{\tilde{\mathbf{x}}}}_i(t) - {\mathbf{q}}_i^\eta \left( {t} \right) \\ &={\mathbf{q}}_i^\eta \left( {t_k^i} \right) - {\rho _i}\left( {t_k^i} \right) {\mathbf{sign}}\left( {{{\mathbf{S}}_i}\left( {t_k^i} \right) } \right) - {\varvec{\Lambda }}{{\mathbf{S}}_i}\left( {t_k^i} \right) - {\hat{\mathbf{d}}}_i\left( {t_k^i} \right) + {{\mathbf{d}}_i}\left( t \right) - {\mathbf{q}}_i^\eta \left( t \right) \\ &={{\mathbf{e}}_i}\left( t \right) - {\rho _i}\left( t \right) {\mathbf{sign}}\left( {{{\mathbf{S}}_i}\left( t \right) } \right) - {\varvec{\Lambda }}{{\mathbf{S}}_i}\left( t \right) + {{\mathbf{d}}_i}\left( t \right) - {\hat{\mathbf{d}}}_i\left( t \right) \end{aligned} \end{aligned}$$

From (20) we can get

22 $$\begin{aligned} \begin{aligned} \dot{V} =\,&{\mathbf{S}}_i^T\left( t \right) {{\dot{\mathbf{S}}}_i}\left( t \right) + \sum \limits _{j = 0}^2 {\left( {{K_j}\left( t \right) - K_j^*} \right) {{\dot{K}}_j}\left( t \right) } \\ = \,&{\mathbf{S}}_i^T\left( t \right) \left[ {{{\mathbf{e}}_i}\left( t \right) - {\rho _i}\left( t \right) {\mathbf{sign}}\left( {{{\mathbf{S}}_i}\left( t \right) } \right) - {\varvec{\Lambda }}{{\mathbf{S}}_i}\left( t \right) + {{\mathbf{d}}_i}\left( t \right) } - {{{{{\hat{\mathbf{d}}}}}}_i}\left( t \right) \right] + \sum \limits _{j = 0}^2 {\left( {{K_j}\left( t \right) - K_j^*} \right) {{\dot{K}}_j}\left( t \right) } \\ \le\,&\left\| {{{\mathbf{S}}_i}\left( t \right) } \right\| \left\| {{{\mathbf{e}}_i}\left( t \right) } \right\| - {\rho _i}\left( t \right) \left\| {{{\mathbf{S}}_i}\left( t \right) } \right\| {\mathbf{sign}}\left( {{{\mathbf{S}}_i}\left( t \right) } \right) - {\varvec{\Lambda }}{\left\| {{{\mathbf{S}}_i}\left( t \right) } \right\| ^{\mathrm{{2}}}} + \left\| {{{\mathbf{S}}_i}\left( t \right) } \right\| \left\| {{{\mathbf{d}}_i}\left( t \right) - {{{{{\hat{\mathbf{d}}}}}}_i}\left( t \right) } \right\| + \sum \limits _{j = 0}^2 {\left( {{K_j}\left( t \right) - K_j^*} \right) {{\dot{K}}_j}\left( t \right) } \\ \le \,&\left\| {{{\mathbf{S}}_i}\left( t \right) } \right\| \left\| {{{\mathbf{e}}_i}\left( t \right) } \right\| - {\rho _i}\left( t \right) \left\| {{{\mathbf{S}}_i}\left( t \right) } \right\| - {\lambda _{\min }}\left( {\varvec{\Lambda }} \right) {\left\| {{{\mathbf{S}}_i}\left( t \right) } \right\| ^{\mathrm{{2}}}} + \left\| {{{\mathbf{S}}_i}\left( t \right) } \right\| \left\| {{{\mathbf{d}}_i}\left( t \right) - {{{{{\hat{\mathbf{d}}}}}}_i}\left( t \right) } \right\| + \sum \limits _{j = 0}^2 {\left( {{K_j}\left( t \right) - K_j^*} \right) {{\dot{K}}_j}\left( t \right) } \end{aligned} \end{aligned}$$

Using (10), (11) and (19), we can get

23 $$\begin{aligned} \begin{aligned} \dot{V} \le&\left\| {{{\mathbf{S}}_i}\left( t \right) } \right\| \left\| {{{\mathbf{e}}_i}\left( t \right) } \right\| - \left( {\sum \limits _{j = 0}^2 {{K_j}\left( t \right) {{\left\| {{{{{{\tilde{\mathbf{x}}}}}}_i}\left( t \right) } \right\| }^j}} + {\tau _i}} \right) \left\| {{{\mathbf{S}}_i}\left( t \right) } \right\| - {\lambda _{\min }}\left( {\varvec{\Lambda }} \right) {\left\| {{{\mathbf{S}}_i}\left( t \right) } \right\| ^{\mathrm{{2}}}} + \left\| {{{\mathbf{S}}_i}\left( t \right) } \right\| \sum \limits _{j = 0}^2 {{K}_j^{*}{{\left\| {{{{{{\tilde{\mathbf{x}}}}}}_i}\left( t \right) } \right\| }^j}} \\&+ \sum \limits _{j = 0}^2 {\left( {{K_j}\left( t \right) - K_j^*} \right) \left( {\left\| {{{\mathbf{S}}_i}\left( t \right) } \right\| {{\left\| {{{{{{\tilde{\mathbf{x}}}}}}_i}\left( t \right) } \right\| }^j} - {\alpha _j}K_j \left( t \right) } \right) } \\ \le&\left( {\left\| {{{\mathbf{e}}_i}\left( t \right) } \right\| {\mathrm{- }}{\tau _i}} \right) \left\| {{{\mathbf{S}}_i}\left( t \right) } \right\| - {\lambda _{\min }}\left( {\varvec{\Lambda }} \right) {\left\| {{{\mathbf{S}}_i}\left( t \right) } \right\| ^{\mathrm{{2}}}} + \sum \limits _{j = 0}^2 {{\alpha _j}\left( {{K_j}\left( t \right) K_j^* - {\alpha _j}K_j^2\left( t \right) } \right) } \end{aligned} \end{aligned}$$

In view of the fact that

24 $$\begin{aligned} \begin{aligned} {K_j}\left( t \right) K_j^* - K_j^2\left( t \right) = - {\left( {\frac{{{K_j}\left( t \right) }}{{\sqrt{2} }} - \frac{{K_j^*}}{{\sqrt{2} }}} \right) ^2} - \frac{{K_j^2\left( t \right) }}{2} + \frac{{K_j^{{*^2}}\left( t \right) }}{2} \le - {\left( {\frac{{{K_j}\left( t \right) }}{{\sqrt{2} }} - \frac{{K_j^*}}{{\sqrt{2} }}} \right) ^2} + \frac{{K_j^{{*^2}}\left( t \right) }}{2} \end{aligned} \end{aligned}$$

hence

25 $$\begin{aligned} \begin{aligned} \dot{V} \le&\left( {\left\| {{{\mathbf{e}}_i}\left( t \right) } \right\| {\mathrm{- }}{\tau _i}} \right) \left\| {{{\mathbf{S}}_i}\left( t \right) } \right\| - {\lambda _{\min }}\left( {\varvec{\Lambda }} \right) {\left\| {{{\mathbf{S}}_i}\left( t \right) } \right\| ^{\mathrm{{2}}}} - \frac{{\mathrm{1}}}{{\mathrm{2}}}\sum \limits _{j = 0}^2 {{\alpha _j}{{\left( {{K_j}\left( t \right) - K_j^*} \right) }^{\mathrm{{2}}}}} {\mathrm{+ }}\frac{{\mathrm{1}}}{{\mathrm{2}}}\sum \limits _{j = 0}^2 {{\alpha _j}K_j^{{*^{\mathrm{{2}}}}}} \end{aligned} \end{aligned}$$

According to the triggering condition given by (16), we can find $\left\| {{{\mathbf{e}}_i}\left( t \right) } \right\| \le {\tau _i}$. Thus, (25) can be further described as

26 $$\begin{aligned} \begin{aligned} \dot{V} \le - \sigma V + \frac{1}{2}\sum \limits _{j = 0}^2 {{\alpha _j}K{{_j^*}^2}\left( t \right) } \end{aligned} \end{aligned}$$

where $\sigma \buildrel \Delta \over = 2{\min _j}\left\{ {{\lambda _{\min }}\left( {\varvec{\Lambda }} \right) ,{{{\alpha _j}} / 2}} \right\} > 0$ is designed by (9) and (11). Defining a scalar $0< \kappa < \sigma$, and (26) simplifies to

27 $$\begin{aligned} \begin{aligned} {\dot{V}} \le - \kappa V - \left( {\sigma - \kappa } \right) V + \frac{1}{2}\sum \limits _{j = 0}^2 {{\alpha _j}K{{_j^*}^2}\left( t \right) } \end{aligned} \end{aligned}$$

Defining a scalar ${\overline{B}} \buildrel \Delta \over = \frac{{\sum \nolimits _{j = 0}^2 {{\alpha _j}K{{_j^*}^2}\left( t \right) } }}{{2\left( {\sigma - \kappa } \right) }}$. It can be seen that $\dot{V} \le - \kappa V$ when $V \ge {\overline{B}}$, so that

28 $$\begin{aligned} \begin{aligned} V \le \max \left\{ { V\left( 0 \right) ,{\overline{B}} } \right\} ,\forall t \ge 0 \end{aligned} \end{aligned}$$

and the Lyapunov function enters in finite time inside the ball defined by ${\overline{B}}$. The definition of the Lyapunov function (20) yields $V \ge \frac{1}{2}{\left\| {{{\mathbf{S}}_i}\left( t \right) } \right\| ^2}$, leading to the ultimate bound (16) on ${\mathbf{S}}_i\left( t \right)$ which is global and uniform as it is independent of initial conditions. The proof of Theorem 1 is completed. $\square$

#### Remark 1

In the algorithm^37,38^, it is assumed that the disturbances are bounded, and the bounds have to be known in advance. The triggering condition still contains the information about the bounds of disturbances, which means that there is a limitation that bound must be known before applying to ensure the stability of the algorithm. However, in the proposed algorithm, the triggering condition (16) does not contain the perturbation boundary. The stability proof process of the closed-loop system does not require any information about the bounds of disturbances.

#### Remark 2

From (17), we could find that the size of the ultimate bound ${\omega }$ depends mainly on ${K}_j^{*}$ (other scalars can be properly chosen). It can be found in (19) that the size of ${K}_j^{*}$ can be effectively reduced by the compensation of the observer, which means that the ultimate bound ${\omega }$ could be very small if a proper disturbance observer is applied.

#### Remark 3

In fact, it is difficult for the disturbance observer to completely observe the real size of the disturbance. The proposed event-triggered ASMC can be effectively coupled with the disturbance observer, so that the remaining perturbation can be effectively handled by the ASMC, and therein lies the key for the proposed algorithm to be robust enough to handle unknown disturbances.

Moreover, the Zeno behavior, *i.e.*, infinite number of triggers in finite time^28^, is one of the main problems to be solved in event-triggered approaches^37^. Due to the utilization of event-triggered strategy in proposed algorithm, it is necessary to analysis on excluding Zeno behavior in the system. Theorem 2 provides a lower bound expression for the inter-event time to ensure that the Zeno behavior can be avoided.

#### Theorem 2

Consider the MAS (2) with control algorithm (14), the communication topology is defined by a connected graph *G* and the triggering condition is given by (20). The Zeno behavior can be avoided in this closed-loop system. Moreover, the inter-event time, *i.e.*, ${T_i} = \left( {t_{k+1}^i - t_k^i} \right)$, implicitly defined by (16), is lower bounded by

29 $$\begin{aligned} \begin{aligned} {T_i} \ge \frac{ {\tau _i} }{{{\gamma _i}}} \end{aligned} \end{aligned}$$

where

$$\begin{aligned} \begin{aligned} {\gamma _i} =\,&{\eta {n^{3 - 2\eta }}\left\| {\Omega \otimes {I_n}} \right\| \left\| {\left( {L + B} \right) \otimes {I_n}} \right\| { {{Q}}^{2\eta - 1}} } + \left( { {P} {n\beta } + \left\| {\varvec{\Lambda }} \right\| } \right) \left[ {\left\| {{\mathbf{q}}_i^\eta \left( {t_k^i} \right) } \right\| + \left\| {{\varvec{\Lambda }}{{\mathbf{S}}_i}\left( {t_k^i} \right) } \right\| } \right. + \left\| {{{{{{\hat{\mathbf{d}}}}}}_i}\left( {t_k^i} \right) } \right\| \\&+ \left. {\left\| {{\rho _i}\left( {t_k^i} \right) {\mathbf{sign}}\left( {{{\mathbf{S}}_i}\left( {t_k^i} \right) } \right) } \right\| + {D_i} + {Q}} \right] + {{\overline{P}}} + {{\overline{D}}} \end{aligned} \end{aligned}$$

and $$\Omega = {\mathrm{diag}}\left[ {{{{\mu _1}} / {{n_1} + 1,{{{\mu _2}} / {{n_2} + 1}}}}...{{{\mu _N}} / {{n_N} + 1}}} \right]$$.

#### Proof

Let ${T_i}$ denote the inter-event time, *i.e.*, the time required for the measurement error to grow to ${\tau _i}$. At $t = t_{k + 1}^i$, *i.e.*, $\left\| {\mathbf{e}_i \left( t \right) } \right\| > {\tau _i}$ the control input is updated, hence the error satisfies $\left\| {\mathbf{e}_i \left( t \right) } \right\| \le {\tau _i}$ and the system waits for the next triggering time. Therefore, the consensus condition holds during the inter-event time. Employing the Lemma 2, we can find $\left\| {{{\dot{\mathbf{q}}}_i}\left( t \right) } \right\| \le \left\| {\Omega \otimes {I_n}}\right\| \left\| {\left( {L + B} \right) \otimes {I_n}} \right\| \left\| {{{\mathbf{q}}_i^\eta }\left( t \right) } \right\| \le \left\| {\Omega \otimes {I_n}} \right\| \left\| {\left( {L + B} \right) \otimes {I_n}} \right\| {n^{1 - \eta }}{\left\| {{\mathbf{q}}_i \left( t \right) } \right\| ^\eta }$, and $\left\| {{\mathbf{q}}_i^{\eta - 1}\left( t \right) } \right\| \le {n^{2 - \eta }}{\left\| {{\mathbf{q}}_i \left( t \right) } \right\| ^{\eta - 1}}$. In practical applications, the system trajectory may deviate from the ideal sliding manifold. But it will remain bounded, depending on ${\tau _i}$.

30 $$\begin{aligned} \begin{aligned} \frac{d}{{dt}}\left\| {{{\mathbf{e}}_i}\left( t \right) } \right\| \le \left\| {\frac{d}{{dt}}{{\mathbf{e}}_i}\left( t \right) } \right\| \le \left\| {\frac{d}{{dt}}\left[ {{\mathbf{q}}_i^\eta \left( t \right) - {\rho _i}\left( t \right) {\mathbf{sign}}\left( {{{\mathbf{S}}_i}\left( t \right) } \right) - {\varvec{\Lambda }}_i{{\mathbf{S}}_i}\left( t \right) - {{\hat{\mathbf{d}}}_i}\left( t \right) } \right] } \right\| \end{aligned} \end{aligned}$$

In order to obtain the derivative of the **sign** function, we use the **tanh** function^45^ to approximate it, *i.e.*, ${\mathbf{sign}}\left( {{{\mathbf{S}}_i}\left( t \right) } \right) \approx {\mathbf{tanh}}\left( {\beta {{\mathbf{S}}_i}\left( t \right) } \right) ,\beta \gg {\mathrm{1}}$. Hence

31 $$\begin{aligned} \begin{aligned} \frac{d}{{dt}} \left\| {{{\mathbf{e}}_i}\left( t \right) } \right\| \le&\left\| {\frac{d}{{dt}}{\mathbf{q}}_i^\eta \left( t \right) } \right\| + \left\| {\frac{d}{{dt}}{\rho _i}\left( t \right) {\mathbf{sign}}\left( {{{\mathbf{S}}_i}\left( t \right) } \right) } \right\| + \left\| {\frac{d}{{dt}}{\varvec{\Lambda }} {{\mathbf{S}}_i}\left( t \right) } \right\| + \left\| {\frac{d}{{dt}}{{\hat{\mathbf{d}}}_i}\left( t \right) } \right\| \\ \le&\eta {n^{3 - 2\eta }}{\left\| {{\mathbf{q}}_i \left( t \right) } \right\| ^{\eta - 1}}\left\| {\Omega \otimes {I_n}} \right\| \left\| {\left( {L + B} \right) \otimes {I_n}} \right\| {\left\| {{\mathbf{q}}_i \left( t \right) } \right\| ^\eta } + {\left\| {{\rho _i}\left( t \right) } \right\| } \left\| {\left[ {{\mathbf{1 - tan}}{{\mathbf{h}}^2}\left( {\beta {{\mathbf{S}}_i}\left( t \right) } \right) } \right] \beta {{\dot{\mathbf{S}}}_i}\left( t \right) } \right\| \\&+ \left\| {{{{\dot{\rho }}}_i}\left( t \right) } \right\| \left\| {{\mathbf{sign}}\left( {{{\mathbf{S}}_i}\left( t \right) } \right) } \right\| + \left\| {\varvec{\Lambda }}_i \right\| \left\| {{{\dot{\mathbf{S}}}_i}\left( t \right) } \right\| + \left\| {{{\dot{\hat{\mathbf{d}}}}_i}\left( t \right) } \right\| \end{aligned} \end{aligned}$$

$\left\| {{{\mathbf{1}}_{n \times n}}{\mathbf{- tan}}{{\mathbf{h}}^2}\left( {\beta {{\mathbf{S}}_i}\left( t \right) } \right) } \right\| \le \left\| {{{\mathbf{1}}_{n \times n}}} \right\| = n$ and using (21), we can find

32 $$\begin{aligned} \begin{aligned} \frac{d}{{dt}} \left\| {{{\mathbf{e}}_i}\left( t \right) } \right\| \le&\eta {n^{3 - 2\eta }}\left\| {\Omega \otimes {I_n}} \right\| \left\| {\left( {L + B} \right) \otimes {I_n}} \right\| {\left\| {{{\mathbf{q}}_i}\left( t \right) } \right\| ^{2\eta - 1}} + \left( {\left\| {{\rho _i}\left( t \right) } \right\| {n\beta } + \left\| {\varvec{\Lambda }} \right\| } \right) \left[ {\left\| {{\mathbf{q}}_i^\eta \left( {t_k^i} \right) } \right\| + \left\| {{\varvec{\Lambda }}{{\mathbf{S}}_i}\left( {t_k^i} \right) } \right\| + \left\| {{{{{{\hat{\mathbf{d}}}}}}_i}\left( {t_k^i} \right) } \right\| } \right. \\&\left. {+ \left\| {{\rho _i}\left( {t_k^i} \right) {\mathbf{sign}}\left( {{{\mathbf{S}}_i}\left( {t_k^i} \right) } \right) } \right\| + \left\| {{{\mathbf{d}}_i}\left( t \right) } \right\| + \left\| {{\mathbf{q}}_i^\eta \left( t \right) } \right\| } \right] + \left\| {{{{{\dot{\rho }}} }_i}\left( t \right) } \right\| \left\| {{\mathbf{sign}}\left( {{{\mathbf{S}}_i}\left( t \right) } \right) } \right\| + \left\| {{{{\dot{{{\hat{\mathbf{d}}}}}}}_i}\left( t \right) } \right\| \end{aligned} \end{aligned}$$

According to the conclusion of Theorem 1, the closed-loop systems (2) are GUUB under the control of algorithm (14), which means that ${\tilde{\mathbf{x}}}_i\left( t \right)$ and ${\mathbf{S}}_i\left( t \right)$ are the bounded signals. In view of (10), we can get that ${{\rho }_i\left( t \right) }$ is the function consisting of the bounded signals, hence ${{\rho }_i\left( t \right) }$ must be bounded. Furthermore, there must exist a positive constant *P* such that $\left\| {{{\rho }_i}\left( t \right) } \right\| \le {P}$. Similarly, there exist positive constants *Q*, ${{\overline{P}}}$ and ${{\overline{D}}}$ that are upper bounds on bounded signals $\left\| {{{\mathbf{q}}_i^\eta }\left( t \right) } \right\|$, $\left\| {{{\dot{\mathbf{\rho }}}_i}\left( t \right) } \right\|$ and $\left\| {{{\dot{\hat{\mathbf{d}}}}_i}\left( t \right) } \right\|$, respectively. It can be found in (3) that $\left\| {{{\mathbf{d}}_i}\left( t \right) } \right\|$ is assumed to be bounded, so there also exist positive ${D_i}$ such that $\left\| {{{\mathbf{d}}_i}\left( t \right) } \right\| \le {D_i}$. Hence,

33 $$\begin{aligned} \begin{aligned} \frac{d}{{dt}} \left\| {{{\mathbf{e}}_i}\left( t \right) } \right\| \le&\eta {n^{3 - 2\eta }}\left\| {\Omega \otimes {I_n}} \right\| \left\| {\left( {L + B} \right) \otimes {I_n}} \right\| { {{Q}}^{2\eta - 1}} + \left( {{P} {n\beta } + \left\| {\varvec{\Lambda }} \right\| } \right) \left[ {\left\| {{\mathbf{q}}_i^\eta \left( {t_k^i} \right) } \right\| + \left\| {{\varvec{\Lambda }}{{\mathbf{S}}_i}\left( {t_k^i} \right) } \right\| + \left\| {{{{{{\hat{\mathbf{d}}}}}}_i}\left( {t_k^i} \right) } \right\| } \right. \\&\left. {+ \left\| {{\rho _i}\left( {t_k^i} \right) {\mathbf{sign}}\left( {{{\mathbf{S}}_i}\left( {t_k^i} \right) } \right) } \right\| + {D_i} + {Q}} \right] + {{\overline{P}}} + {{\overline{D}}}\\ \end{aligned} \end{aligned}$$

With the initial condition $$\left\| {{{\mathbf{e}}_i}\left( t \right) } \right\| = 
0$$, $$\left\| {{{\mathbf{e}}_i}\left( t \right) } \right\|$$ can be calculated as follows

34 $$\begin{aligned} \begin{aligned} \left\| {{{\mathbf{e}}_i}\left( t \right) } \right\| \le&\left( {t - t_k^i} \right) \left\{ {\eta {n^{3 - 2\eta }}\left\| {\Omega \otimes {I_n}} \right\| \left\| {\left( {L + B} \right) \otimes {I_n}} \right\| { {{Q}}^{2\eta - 1}} } \right. + \left( { {P} {n\beta } + \left\| {\varvec{\Lambda }} \right\| } \right) \left[ {\left\| {{\mathbf{q}}_i^\eta \left( {t_k^i} \right) } \right\| + \left\| {{\varvec{\Lambda }}{{\mathbf{S}}_i}\left( {t_k^i} \right) } \right\| } \right. + \left\| {{{{{{\hat{\mathbf{d}}}}}}_i}\left( {t_k^i} \right) } \right\| \\&\left. { + \left. {\left\| {{\rho _i}\left( {t_k^i} \right) {\mathbf{sign}}\left( {{{\mathbf{S}}_i}\left( {t_k^i} \right) } \right) } \right\| + {D_i} + {Q}} \right] + {{\overline{P}}} + {{\overline{D}}} } \right\} \end{aligned} \end{aligned}$$

When $\left\| {{{\mathbf{e}}_i}\left( t \right) } \right\| > {\tau _i}$, the event is triggered, and we know $\left( {t - t_k^i} \right) \le {T_i}$. Hence,

35 $$\begin{aligned} \begin{aligned} {\tau _i} < \left\| {{{\mathbf{e}}_i}\left( t \right) } \right\| \le&{T_i}\left\{ {\eta {n^{3 - 2\eta }}\left\| {\Omega \otimes {I_n}} \right\| \left\| {\left( {L + B} \right) \otimes {I_n}} \right\| { {{Q}}^{2\eta - 1}} } \right. + \left( { {P} {n\beta } + \left\| {\varvec{\Lambda }} \right\| } \right) \left[ {\left\| {{\mathbf{q}}_i^\eta \left( {t_k^i} \right) } \right\| + \left\| {{\varvec{\Lambda }}{{\mathbf{S}}_i}\left( {t_k^i} \right) } \right\| } \right. + \left\| {{{{{{\hat{\mathbf{d}}}}}}_i}\left( {t_k^i} \right) } \right\| \\&\left. { + \left. {\left\| {{\rho _i}\left( {t_k^i} \right) {\mathbf{sign}}\left( {{{\mathbf{S}}_i}\left( {t_k^i} \right) } \right) } \right\| + {D_i} + {Q}} \right] + {{\overline{P}}} + {{\overline{D}}} } \right\} \end{aligned} \end{aligned}$$

Therefore, we can ensure that the lower bound of the inter-event time given by (29) is a strictly positive value. The proof of Theorem 2 is completed. $\square$

Moreover, the **tanh** function can be used instead of the **sign** function to reduce the chattering inherent in SMC^46^.

## Results

### Numerical simulations

In this section, we consider a leader-follower MAS consisting of some moving point masses, each of which is modeled as a MIMO single integral system, and the problem is considered in the 3-D plane. We assume that the leader can provide the ideal reference trajectory, and the follower is subject to unknown disturbances. According to (1) and (2), the dynamics of the agent *i* relative to the leader can be described as

36 $$\begin{aligned} \begin{aligned} {{\dot{\tilde{\mathbf{x}}}}}_i(t) = {\tilde{\mathbf{u}}}_i\left( t \right) + {\mathbf{d}}_i\left( t \right) , i = 1,\ldots ,N \end{aligned} \end{aligned}$$

where ${\tilde{\mathbf{x}} }_i\left( t \right) = {\mathbf{x}}_i\left( t \right) - {\mathbf{x}}_0\left( t \right) + {\varvec{\delta } }_i$. ${{{\mathbf{x}}}_i\left( t \right) } \in {\mathbb {R}}^3$ contains three channels as a position vector in $\left( {x,y,z} \right)$ coordinates. ${{{\mathbf{x}}}_0\left( t \right) } \in {\mathbb {R}}^3$ represent the position vector of the leader, which is considered an ideal reference trajectory without any disturbance. ${{\varvec{\delta } }_i} = {\left( {{\delta }_i^x}, {{\delta }_i^y},{{\delta }_i^z} \right) }^T$ is the desired position deviation of the ${i^{th}}$ follower from the leader. ${\tilde{\mathbf{u}} }_i\left( t \right) \in {\mathbb {R}}^3$ and ${{{\mathbf{d}}}_i\left( t \right) } \in {\mathbb {R}}^3$ represent the control input and the unknown disturbances for the ${i^{th}}$ follower, respectively.

**Figure 2:**
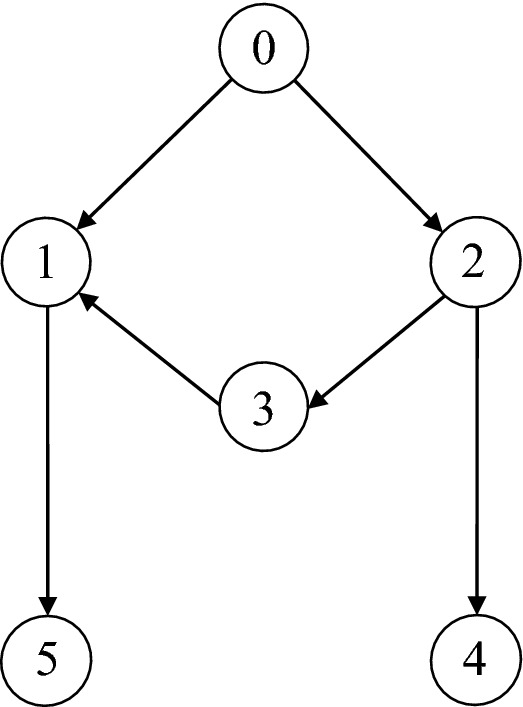
Communication network in the simulation.

Consider the MAS with $N+1$ members (the parameter $N = 5$), as shown in the Fig. 2, one of which marks the leader and the others as followers. The connected communication topology graph and its parameters are as follows

$$\begin{aligned} L = \left( {\begin{array}{*{20}{c}} 1&{}0&{}{ - 1}&{}0&{}0\\ 0&{}0&{}0&{}0&{}0\\ 0&{}{ - 1}&{}{1}&{}0&{}0\\ 0&{}0&{}{ - 1}&{}{1}&{}0\\ { - 1}&{}0&{}0&{}0&{}{1} \end{array}} \right) , \quad B = \left( {\begin{array}{*{20}{c}} 1&{}0&{}0&{}0&{}0\\ 0&{}1&{}0&{}0&{}0\\ 0&{}0&{}0&{}0&{}0\\ 0&{}0&{}0&{}0&{}0\\ 0&{}0&{}0&{}0&{}0 \end{array}} \right) \end{aligned}$$

The event-triggered ASMC based consensus algorithm parameters are chosen as follows: ${{\tau }_i} = 0.35$, ${{\varvec{\Lambda }}} = {\mathrm{diag}}\left[ {0.08,0.08,0.08} \right]$, ${\mu _i} = 0.1$, $\eta = {5 / 7}$ and the adaptive switching gain parameters are chosen as: $\alpha _0 = \alpha _1 = \alpha _2 = 0.1$ and the initial value ${K}_0 \left( 0 \right) = 0.25$, ${K}_1 \left( 0 \right) = 0.05$, ${K}_2 \left( 0 \right) = 0.01$. The value of adaptive multivariable disturbance observer parameters are as follows: $c_1 = c_2 = 0.3,c_3 = c_4 = 0.5$, $\alpha _g = 0.1$. The initial value for adaptive gain is chosen as ${G}\left( 0 \right) = 0.25$. In practice, ${{\mathbf{E}}_i}\left( t \right)$ in (4) cannot be zero exactly. The leader’s trajectory and desired position deviations are chosen as ${{\mathbf{x}}_0}\left( t \right) = {\left( {\sin \left( {0.25t} \right) ,\cos \left( {0.25t} \right) ,0.1t } \right) ^T}$, ${{\varvec{\delta }}_1}\left( t \right) = {\left( {-0.5,0.5,0} \right) ^T}$, ${{\varvec{\delta }}_2}\left( t \right) = {\left( {0.5, 0.5,0} \right) ^T}$, ${{\varvec{\delta }}_3}\left( t \right) = {\left( {0,0,0.5} \right) ^T}$, ${{\varvec{\delta }}_4}\left( t \right) = {\left( {0.5,-0.5,0} \right) ^T}$ and ${{\varvec{\delta }}_5}\left( t \right) = {\left( {-0.5,-0.5,0} \right) ^T}$. The initial positions ${{{\mathbf{x}}}_i\left( 0 \right) } = {\left( {0,0,0} \right) ^T}$. Inspired by Zhang^47^ and Xiao^48^, the unknown disturbances in the simulation are more suitable to be assumed as

37 $$\begin{aligned} \begin{aligned} {{\mathbf{d}}_i}\left( t \right) = {0.25}\left[ {\begin{array}{*{20}{c}} {\cos \left( {0.5\pi t} \right) + \sin \left( {0.3\pi t} \right) }\\ {\sin \left( {0.5\pi t} \right) + \cos \left( {0.3\pi t} \right) }\\ {\sin \left( {0.5\pi t} \right) + \sin \left( {0.3\pi t} \right) } \end{array}} \right] \end{aligned} \end{aligned}$$

The simulations are completed in MATLAB/Simulink environment with a sampling time of 1 ms.

**Figure 3:**
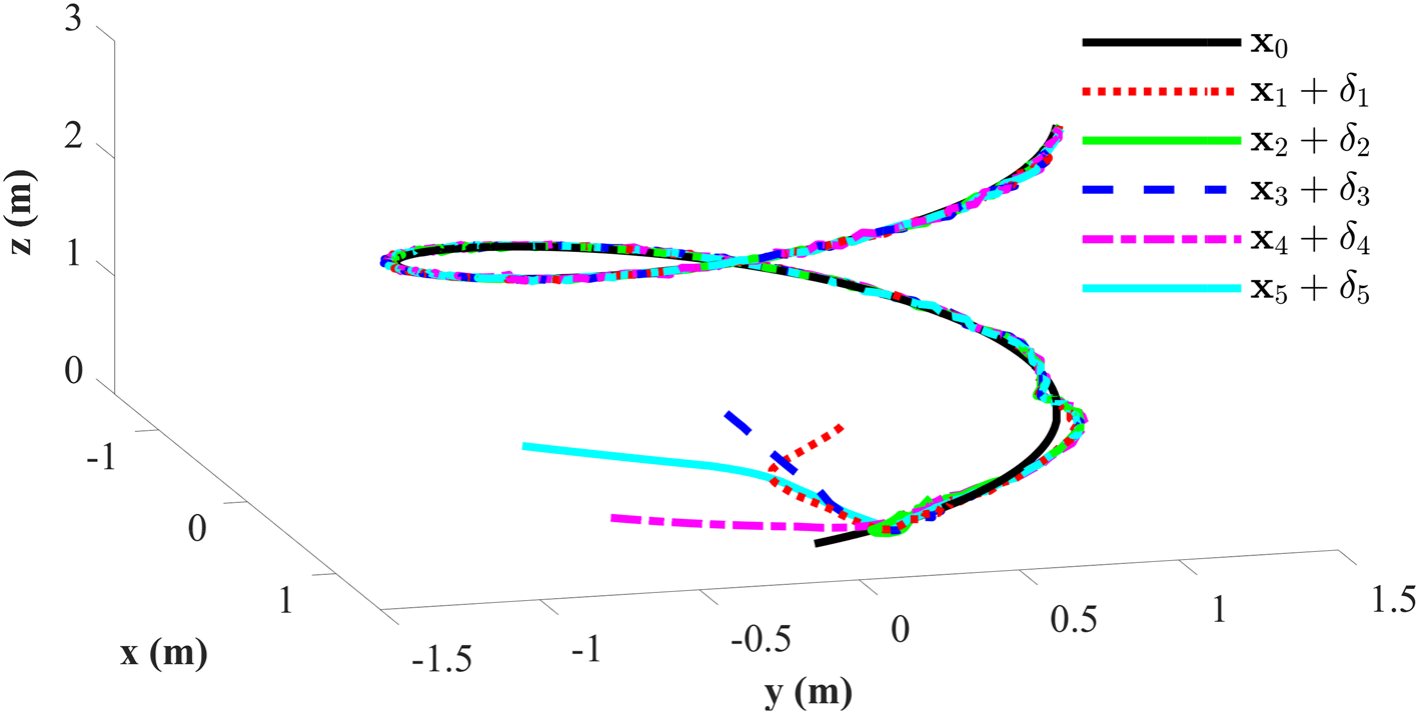
Simulation results: Trajectories of the multiagent system in $\left( {x,y,z} \right)$ coordinate.

**Figure 4:**
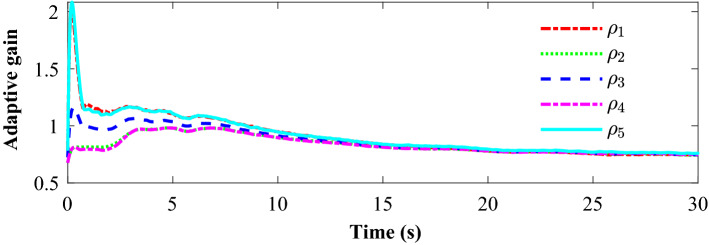
Simulation results: The adaptive switching gain for different agnets.

**Figure 5:**
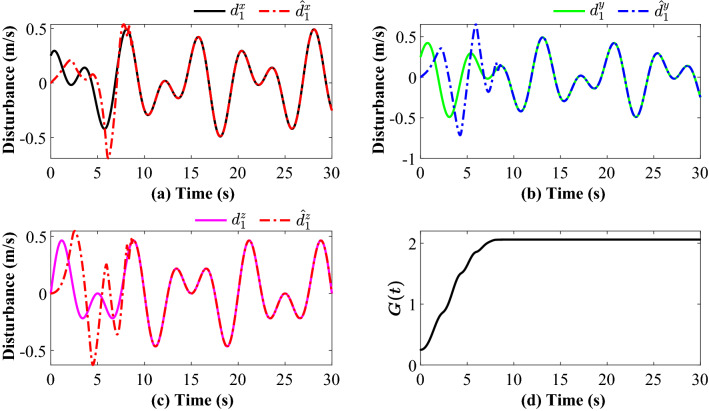
Simulation result: (**a**), (**b**), (**c**) and (**d**) represent X, Y, Z disturbance tracking and ${G}\left( t \right)$ in follower 1, respectively.

**Figure 6:**
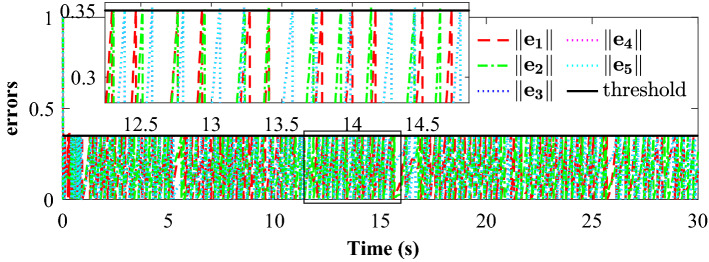
Simulation results: The event-triggered measurement error for different agents.

**Figure 7:**
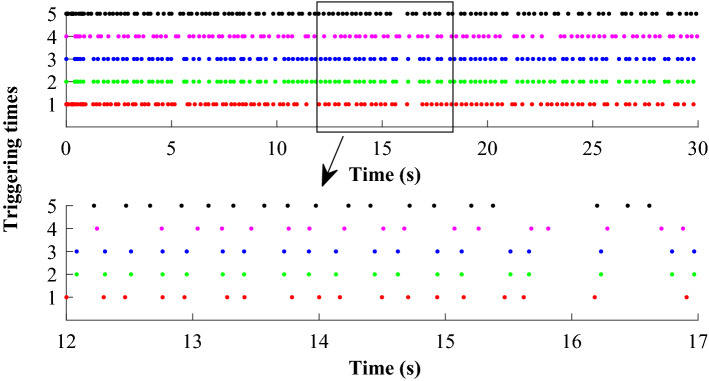
Simulation results: The triggering times for different agents (The numbers 1, 2, 3, 4, and 5 on the y-axis represent agent1, 2, 3, 4, and 5, respectively).

**Figure 8:**
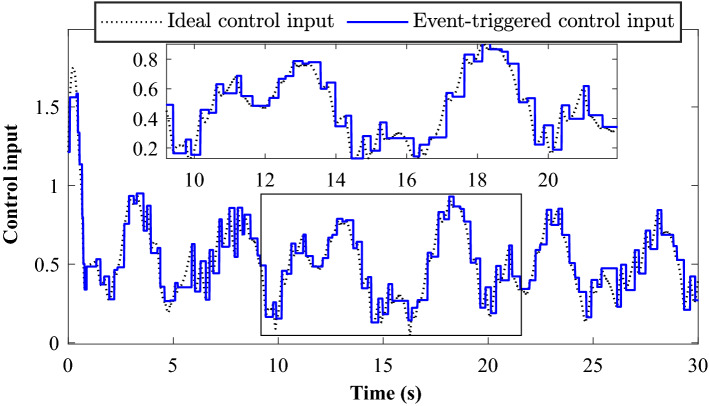
Simulation results: The event-triggered control input and the ideal control input of follower 3 (The event-triggered control input means $\left\| {{{\tilde{\mathbf{u}}}_i}\left( t \right) } \right\|$, where ${{\tilde{\mathbf{u}}}_i}\left( t \right)$ is described in (14) with event-triggered strategy, the ideal control input means $\left\| {{{\tilde{\mathbf{u}}}_i}\left( t \right) } \right\|$, where ${{\tilde{\mathbf{u}}}_i}\left( t \right)$ is described in (13) without event-triggered strategy).

**Figure 9:**
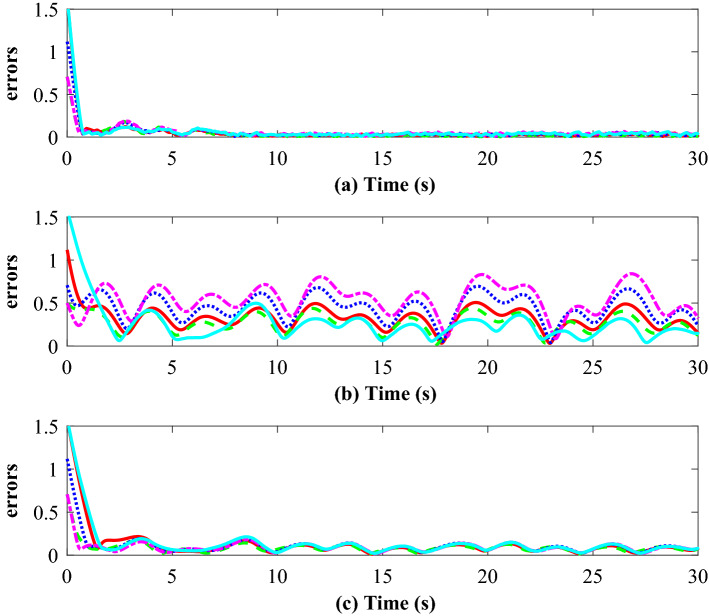
Simulation results: (**a**), (**b**) and (**c**) represents the consensus errors (m) for different agents under the control of proposed algorithm, algorithm^27^ and algorithm^37^, respectively.

Tracking performance of all the agent compared to the desired trajectory is illustrated in Fig. 3. It can be found that the followers can quickly reach a consensus with the leader and converge to the desired position. Figure 4 depicts the adaptive switching gain. The adaptive multivariable disturbance observer of follower 1 is plotted in Fig. 5. We can find that the disturbance observer can effectively track the disturbances in the system, and the adaptive gain $G\left( t \right)$ can also be effectively adjusted according to the state estimation error. In order to exclude Zeno behavior, a non-zero positive trigger threshold ${\tau _i}$ is designed in the proposed method. Therefore, the event-triggered measurement error for different agents are undulant from 0 to ${\tau _i}$, as shown in Fig. 6. The control input of each agent is updated only when the triggering condition is satisfied, *i.e.*, $\left\| {{{\textbf {e}}}_i \left( t \right) } \right\| > {\tau _i}$, and remains constant through the ZOH during the inter-event time. The triggering times of the agents in the simulation are shown in Fig. 7. Figure 8 depicts the comparison between ideal control input and event-triggered control input of follower 3. The consensus errors for different agents $\left\| {{{{\tilde{\mathbf{x}}}}_i}\left( t \right) } \right\|$ are plotted in Fig. 9a. It can be found in Table 1 that the inter-event time satisfies the lower bound condition, which means that Zeno behavior can be avoided. The triggered times are much less than the total sampling times, which means that control efficiency is greatly improved.

To compare the effectiveness and robustness of the proposed algorithm with the existing algorithms, we repeat the simulations based on a traditional event-triggered consensus algorithm proposed by Zhu^27^, which is defined as ${{{\tilde{\mathbf{u}}}}_i}\left( t \right) = {\gamma _i}{\mathbf{q}}_i^\alpha \left( t_k^i \right)$, where the scalar ${\gamma _i} > 0$. The triggering condition is designed as $\left\| {{{\mathbf{e}}_i}\left( t \right) } \right\| \ge {\left( {\frac{{{\varepsilon _i}}}{{{n^{{{\left( {1 - \beta } \right) } / 2}}}}}} \right) ^{{1 / \beta }}}{\left\| {{{\mathbf{q}}_i}\left( t \right) } \right\| ^{{\alpha / \beta }}}$, where ${{\mathbf{e}}_i}\left( t \right) = {\left( {{\mathbf{q}}_i^\alpha \left( {t_k^i} \right) - {\mathbf{q}}_i^\alpha \left( t \right) } \right) ^{{1 / \beta }}}$, and $\alpha \in \left( 0 \right. ,\left. 1 \right)$,$\beta \in \left( 0 \right. ,\left. 1 \right]$. Figure 9 (b) depicts the tracking performance based on the algorithm^27^, and it can be found that the consensus errors are unacceptably high. This means that it may not be suitable for application in the MAS with disturbances, which cannot withstand the uncertainties/external disturbances in the system.

In addition, we repeat the simulation using a traditional SMC based event-triggered consensus algorithm proposed by Nair^37^, which is defined as ${{\tilde{\mathbf{u}}}_i}\left( t \right) = {\mathbf{q}}_i^\eta \left( t \right) - {{\mathbf{K}}_1}{\mathbf{sign}}\left( {{{\mathbf{S}}_i}\left( t \right) } \right) - {{\mathbf{K}}_2}{{\mathbf{S}}_i}\left( t \right)$ and the triggering condition is as follows $\left\| {{{\mathbf{e}}_i}\left( t \right) } \right\| \le {\rho _i}$, where ${{\mathbf{e}}_i}\left( t \right) = {\mathbf{q}}_i^\eta \left( t_k^i \right) - {{\mathbf{K}}_1}{\mathbf{sign}}\left( {{{\mathbf{S}}_i}\left( t_k^i \right) } \right) - {{\mathbf{K}}_2}{{\mathbf{S}}_i}\left( t_k^i \right) - \left[ {\mathbf{q}}_i^\eta \left( t \right) - {{\mathbf{K}}_1}{\mathbf{sign}}\left( {{{\mathbf{S}}_i}\left( t \right) } \right) - {{\mathbf{K}}_2}{{\mathbf{S}}_i}\left( t \right) \right]$, ${\rho _i} < {\lambda _{\min }}\left( {{{\mathbf{K}}_1}} \right) - {D_i}$ and ${\lambda _{\min }}\left( {{{\mathbf{K}}_1}} \right) > {D_i}$. Figure 9c depicts the consensus errors based on the algorithm^37^. Comparing Fig. 9a–c, we could see that it can handle the uncertainties/external disturbances in the system more effectively than algorithm^27^, and the control performance is very close to that of the proposed algorithm. However, there has a limitation that algorithm^37^’s triggering condition still^37^ contains the bounds of disturbances, when compared with the proposed algorithm. In many application scenarios, the bounds can not be unknown in advance, so it is difficult to completely ensure the stability.

Finally, some important data for the MAS in the simulation, such as the triggered times, the maximum inter-event time, the minimum inter-event time and the average consensus error, are detailed compared in Table 1. It can be found that compared with existing methods, the proposed algorithm has better performance in the triggered times, the trigger interval and the control performance. Therefore, based on the theoretical analysis, numerical simulation and comparing with existing methods, it can be concluded that the proposed event-triggered ASMC algorithm can make up for the shortcomings of existing ones and effectively solve the distributed consensus control problem of the MIMO MASs with unknown disturbances in a leader-follower framework.

**Table 1:** Some statistics of the multiagent system in the simulation, total sampling times are 30001 (Max. inter-evnet, Min. inter-event and Mean. consensus error represents the maximum inter-evnet time (s), the minimum inter-evnet time (s) and the average consensus error (m) in the simulation, respectively).

Algorithm	Agent No.	Triggered times	Max. inter-evnet	Min. inter-event	Mean. consensus error
Proposed algorithm	Follower 1	128	0.727	0.018	0.0512
	Follower 2	112	0.617	0.060	0.0370
	Follower 3	118	0.766	0.053	0.0513
	Follower 4	105	0.894	0.063	0.0537
	Follower 5	129	0.824	0.017	0.0593
Algorithm^27^	Follower 1	392	0.466	0.005	0.3199
	Follower 2	355	0.376	0.006	0.2691
	Follower 3	335	0.456	0.006	0.4388
	Follower 4	297	0.854	0.015	0.5361
	Follower 5	295	0.598	0.013	0.4591
Algorithm^37^	Follower 1	336	0.401	0.025	0.1185
	Follower 2	351	0.352	0.015	0.0854
	Follower 3	324	0.393	0.020	0.1065
	Follower 4	325	0.409	0.014	0.0931
	Follower 5	331	0.407	0.081	0.1286

### Application to a group of small UAVs

**Figure 10:**
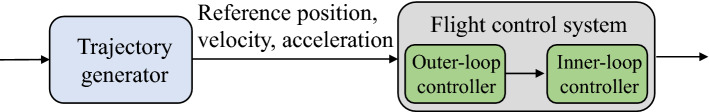
Hierarchical framework for trajectory tracking.

Currently, most autonomous unmanned aerial systems have a hierarchical framework for tracking a trajectory, as shown in Fig. 10. Different algorithms are used in the upper-level trajectory generator module to provide reference information such as position, velocity and acceleration to the lower-level flight control system. The flight control system generally consists of two layers, *i.e.*, the inner-loop and outer-loop controller. The purpose of the inner-loop controller is to stabilize the attitude of the unmanned vehicle. A properly designed inner-loop controller would make the vehicle dynamics behave like a point mass, while it can be controlled by an outer-loop controller to track the reference generated by the trajectory generator^20^. This hierarchical framework promotes us to focus on the trajectory generation algorithm without paying too much attention to the flight control system. Therefore, in this subsection, we can conduct real-time flight tests using a MAS consisting of small UAVs with flight control system to verify whether the proposed algorithm can provide robust reference for the MAS to achieve consensus-based formation.

**Figure 11:**
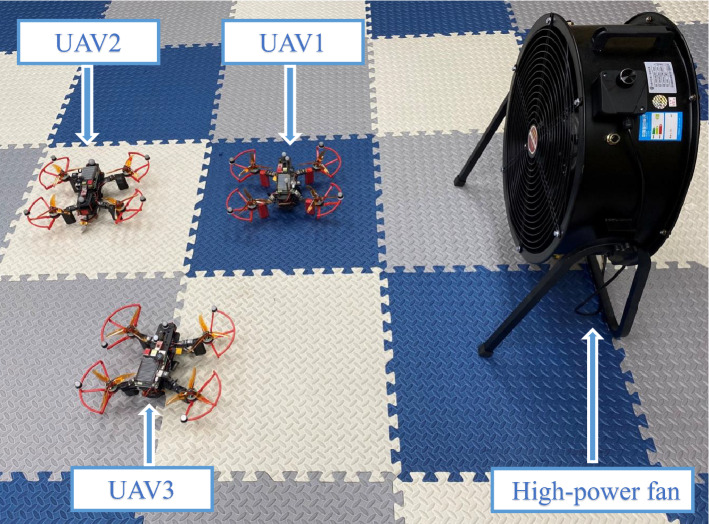
Small UAVs experimental platform.

The real-time experiments are done using a group of small UAVs with 4 members labeled 0, 1, 2 and 3. The UAV0 is virtual running in the ground station and provides ideal reference trajectory. Three real quadrotors (UAV1, UAV2 and UAV3) with the same parameters, as shown in Fig. 11. The real quadrotor, mainly consists of Pixhawk 4 mini flight control module with a properly designed flight control system^49^, power system with T-Motor’s V2207-1950kv motor^50^ and qav250 frame, equipped with an on-board computer running Ubuntu 18.04 operating system, has a total weight about 843g. Although the self-assembled UAV may have some jitter under the control of Pixhawk 4 mini flight control module and cannot be modeled as a perfect point mass, it can be considered as a point mass with unmodeled system dynamics. Furthermore, the unknown disturbances for the following UAVs is also influenced by wind perturbations generated by high-power fan, shown in Fig. 11. The consensus-based formation tracking problem of small UAVs based on leader-follower framework is considered in a 3-D plane. So, their dynamics can be described by (36). Therefore, we can use the proposed event-triggered ASMC based consensus algorithm ${\tilde{\mathbf{u}}}_i\left( t \right)$ described by (14) to generate reference velocity for the flight control system to track. This verifies whether the proposed algorithm could provide sufficiently robust reference to achieve consensus-based formations for small UAVs subject to the unknown disturbances.

**Figure 12:**
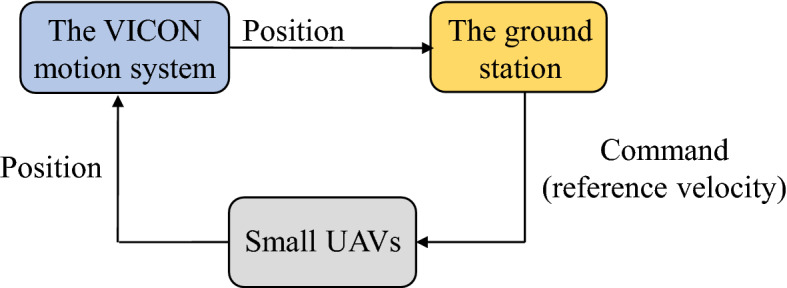
Experimental setup for real flight tests.

**Figure 13:**
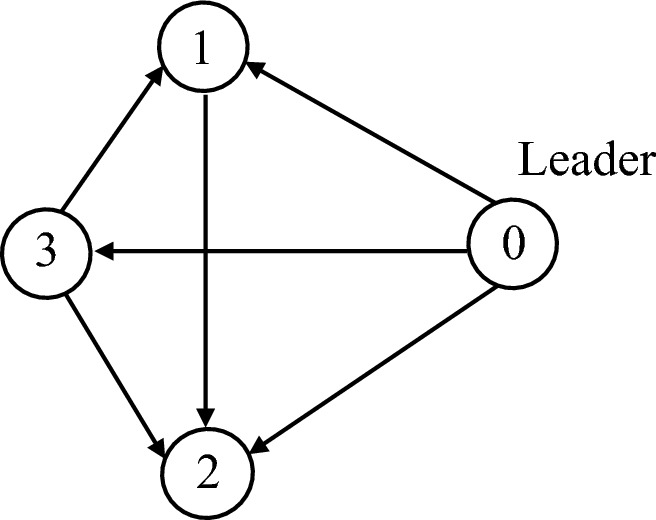
Communication network in the experiment.

The real flight tests are conducted in the Vicon room, where is equipped with a low-cost Wi-Fi local area network. Fig. 12 indicate experimental setup for the real flight tests with a group of small UAVs. Firstly, The Vicon motion system^51^, captures the position information of the quadrotor equipped with identifiable markers, and the position data are sent to the ground station through the Robot Operating System (ROS). At the ground station, the leader UAV0 is simulated to fly and provide an ideal reference trajectory. Our algorithm, running in the ground station, calculates the reference velocity for each follower and publishes it to the network via Wi-Fi, based on the reference trajectory and the captured position data. The quadrotor subscribes the needed reference velocity via Wi-Fi, and to enable the built-in flight controller to track the reference velocity generated by the proposed algorithm. The communication network between UAVs is shown in Fig. 13, and its parameters are as follows

$$\begin{aligned} L = \left( {\begin{array}{*{20}{c}} 1&{}0&{}{ - 1}\\ { - 1}&{}2&{}{ - 1}\\ 0&{}0&{}0 \end{array}} \right) , \quad B = \left( {\begin{array}{*{20}{c}} 1&{}0&{}0\\ 0&{}1&{}0\\ 0&{}0&{}1 \end{array}} \right) \end{aligned}$$

In the experiment, the event-triggered ASMC based consensus algorithm parameters are chosen as follows ${{\tau }_i} = 0.05$, ${{\varvec{\Lambda }}} = {\mathrm{diag}}\left[ {0.08,0.08,0.08} \right]$, ${\mu _i} = 0.1$, $\eta = {5 / 7}$ and the adaptive switching gain parameters are chosen as $\alpha _0 = \alpha _1 = \alpha _2 = 0.1$ and the initial value ${K}_0 \left( 0 \right) = 1$, ${K}_1 \left( 0 \right) = {K}_2 \left( 0 \right) = 0.1$. The value of adaptive multivariable disturbance observer parameters are as follows $c_1 = c_2 = 0.1$, $c_3 = c_4 = 0.3$, $\alpha _g = 0.1$. The initial value for adaptive gain is chosen as ${G}\left( 0 \right) = 0.1$. In practice, ${{\mathbf{E}}_i}\left( t \right)$ in (4) cannot be zero exactly. The desired position deviation is chosen as ${{\varvec{\delta }}_1}\left( t \right) = {\left( {0,1.75,0} \right) ^T}$, ${{\varvec{\delta }}_2}\left( t \right) = {\left( {0, -0.75,0} \right) ^T}$, ${{\varvec{\delta }}_3}\left( t \right) = {\left( {1,0.5,0} \right) ^T}$. The UAV0 moves from position $\left( { - 2.5,0,0.5} \right)$ to $\left( {1.5,0,0.5} \right)$ at a speed of 2 m/s. The high-power fan is placed at a position $\left( {{\mathrm{0}},{\mathrm{3}}{\mathrm{.0}},0} \right)$ in the room. During the flight, the UAVs first take off to an altitude of 0.5 m and then the proposed consensus control algorithm starts to work, then hover and form the desired formation, execute the leader’s trajectory and hover again, finally they descend to the ground.

**Figure 14:**
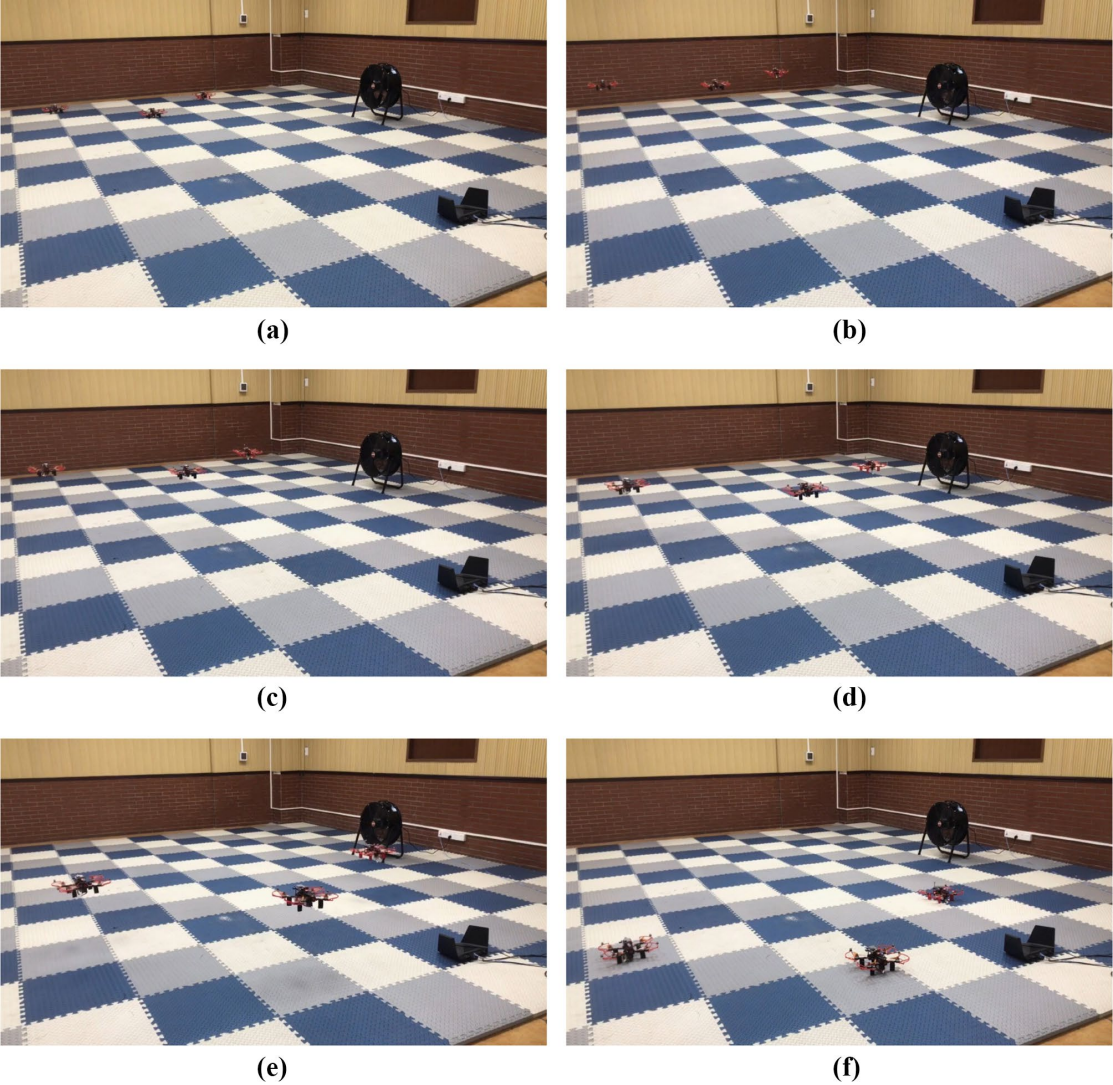
The experimental video image sequences (**a**)–(**f**) of a group of small UAVs flying in formation when faced with disturbances.

**Figure 15:**
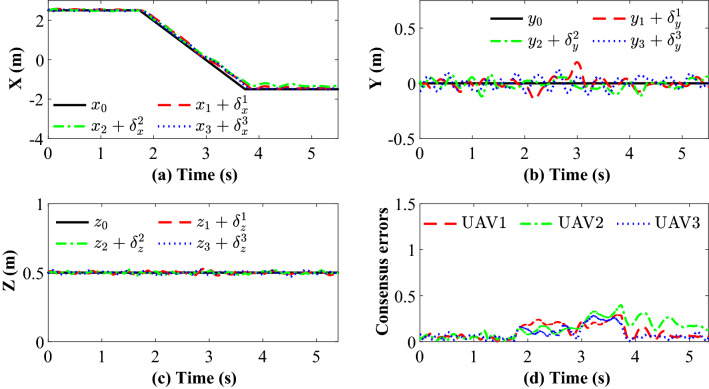
Experimental results: (**a**), (**b**), (**c**) and (**d**) represent X, Y, Z position trajectory and consensus error (m), respectively.

**Figure 16:**
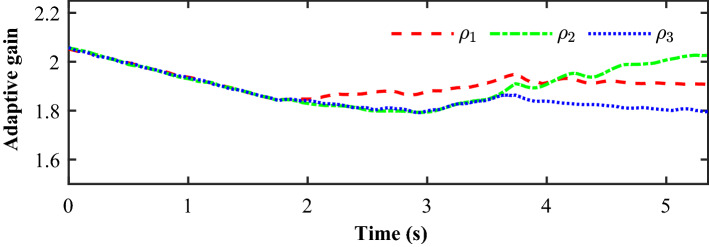
Experimental results: The adaptive switching gain.

**Figure 17:**
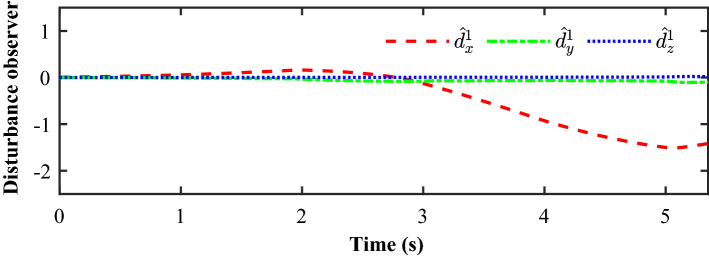
Experimental results: The Estimation results of UAV1.

**Figure 18:**
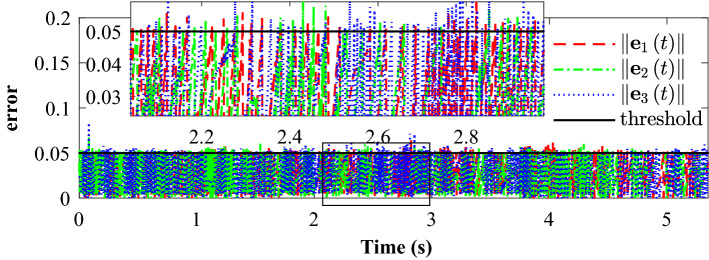
Experimental results: Measurement error for followers.

**Figure 19:**
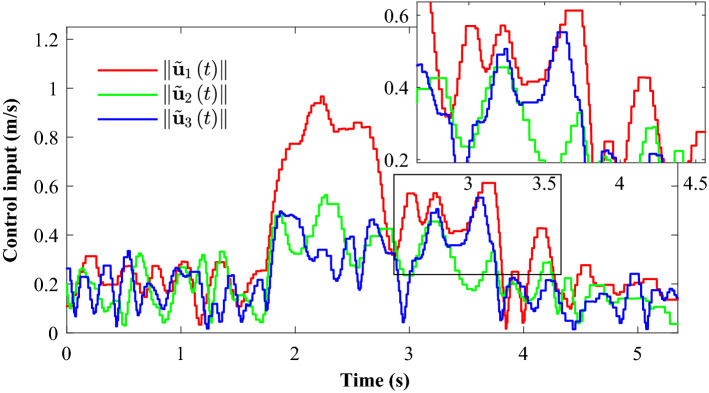
Experimental results: Velocity (norm of the control input: $\left\| {{\tilde{\mathbf{u}} }_i\left( t \right) } \right\|$.

Figure 14 completely describes the experimental process of the small UAVs taking off, flying in formation facing disturbance and finally landing. From the tracking performance of the UAVs given in Fig. 15, it is clear that the followers are reaching into consensus with the leader and converge to desired position deviation, in spite of unknown disturbances. Figure 16 depicts the adaptive switching gain. The disturbance estimation results of UAV1 are plotted in Fig. 17. The measurement error given in Fig. 18 shows that the control is triggered as long as the trigger condition is violated. Figure 19 shows the control input $\left\| {{{{\tilde{\mathbf{u}}}}_i}\left( t \right) } \right\|$ (velocity).

**Figure 20:**
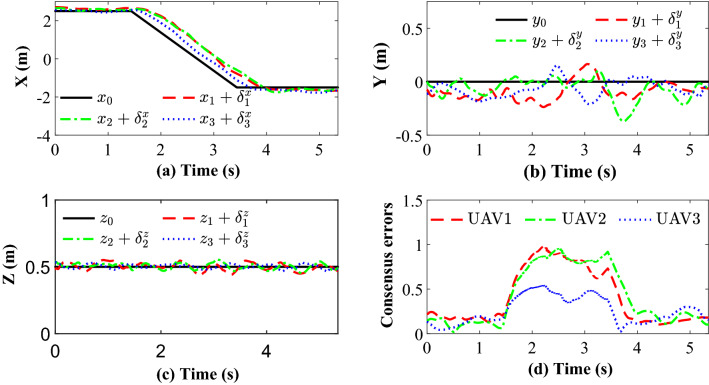
Experimental results based on algorithm^37^: (**a**), (**b**), (**c**) and (**d**) represent X, Y, Z position trajectory and consensus error (m), respectively.

In order to highlight the application value of the proposed algorithm, we also repeat the experiment using the algorithm^37^. Figure 20 depicts the tracking performance based on the algorithm^37^. We can find that consensus can be reached when the perturbation is weak, while the consensus error is unacceptably high when the perturbation is strong. The major reason for this phenomenon is that the trigger condition of the algorithm^37^ is that the value of ${D_i}$ needs to be known in advance, but ${D_i}$ is unknown in the above experimental scenario. This makes it difficult to choose the values of ${{\mathbf{K}}_1}$ and ${\rho _i}$ reasonably so that the algorithm can have excellent control performance in this experiment as well. Although, we can choose the maximum possible ${{\mathbf{K}}_1}$ and the minimum possible ${\rho _i}$ to make the algorithm^37^ handle most of the disturbances. This would make the event-triggered controller very sensitive, even in the absence of disturbances, which is contrary to the motivation for applying the event-triggered strategy. In contrast to the algorithm^37^, the proposed algorithm is able to adaptively adjust the switching gain according to the state to overcome unknown perturbations without any prior knowledge of the bounds of the disturbances. It confirms the advantages of event-triggered ASMC in this type of MAS.

## Conclusion

In this paper, the distributed consensus control problem of the MIMO MASs with unknown disturbances in a leader-follower-based framework has been thoroughly investigated, and a novel robust distributed consensus control scheme based on event-triggered ASMC has been proposed and verified. The advantages of the proposed scheme are that it does not require any information on the bounds of the disturbance. All the results have been validated by numerical simulations and real-time experiments using a group of small UAVs. The results have shown that the proposed algorithm is robust enough to handle the unknown disturbances in the system and avoid the Zeno behavior simultaneously. The results are also compared with existing methods, showing that it has more application value. Future work will focus on the communication between agents and design an event-triggered broadcast mechanism to reduce communication cost.

## Data Availability

The complete experiment data is available by contacting the corresponding author.
